# Evaluation of Xpert MTB/XDR and Deeplex Myc‐TB for the Rapid Detection of Drug Resistance in *Mycobacterium tuberculosis*

**DOI:** 10.1155/ijm/8659735

**Published:** 2026-04-24

**Authors:** Mirae Park, Priya Solanki, Timothy Daniel McHugh, Onn Min Kon, Giovanni Satta

**Affiliations:** ^1^ Department of Respiratory Medicine, St Mary’s Hospital, Imperial College Healthcare NHS Trust, London, UK, nhs.uk; ^2^ National Heart and Lung Institute, Imperial College London, London, UK, imperial.ac.uk; ^3^ UCL-TB and Centre for Clinical Microbiology, Division of Infection & Immunity, Royal Free Campus, University College London, London, UK, ucl.ac.uk

## Abstract

**Objectives:**

Rapid identification of drug‐resistant tuberculosis remains critical for the initiation of appropriate treatment regimens, improved success rates and reduction in the development of drug resistance. In this study, we compared the diagnostic performance of two novel diagnostic tests, Xpert MTB/XDR and Deeplex Myc‐TB (both CE marked).

**Methods:**

Twenty clinical isolates of *Mycobacterium tuberculosis* (MTB) with known drug resistance patterns were used to determine the concordance/discordance of the two rapid platforms when performing drug susceptibility testing. The limit of detection for MTB, the detection of coinfection/cross‐reactivity with *Mycobacterium abscessus* (Mab) and the detection of heteroresistance using mixtures of wild type MTB and drug‐resistant MTB were also evaluated.

**Results:**

Xpert MTB/XDR had a total concordance of 68% and Deeplex Myc‐TB had a total concordance of 100% when compared to WGS and phenotypic drug susceptibility testing. Xpert TB/XDR had a lower limit of detection for identifying MTB at 10^3 CFU/mL, whereas Deeplex Myc‐TB required at least 10^4 CFU/mL. Both Xpert MTB/XDR and Deeplex Myc‐TB were able to detect the wild type MTB without any cross‐reactivity with the drug‐resistant Mab. For heteroresistance detection, Deeplex Myc‐TB was able to detect down to 1% admix of the resistant isolates (1 out of 4), whilst Xpert MTB/XDR was only able to detect down to 10% in 1 out of 4 mixtures of isolates tested.

**Conclusion:**

Both platforms represent an attractive option for the rapid detection of drug resistance and heteroresistance contributing to the management of complex cases, but further studies are needed to assess their real clinical impact.

## 1. Introduction

Drug‐resistant tuberculosis (TB) continues to be growing concern worldwide, with the global rates of multidrug‐resistant (MDR) and rifampicin‐resistant TB (RR‐TB) increasing by 3.1% since 2020 to 450,000 incident cases [1]. Data from the World Health Organization (WHO) showed that 33,520 TB cases were reported in the European Union/European Economic Area (EU/EEA) in 2021, with this number increasing to over 166,000 (new and relapsed TB cases) when considering the wider WHO European Region. The burden of RR‐TB is also estimated to have increased, with 1 in 3 cases of pulmonary TB in the region now resistant to rifampicin. Only 62% of pulmonary TB patients were aware of their rifampicin resistance status, and around 30% of rifampicin‐resistant patients were also resistant to fluoroquinolones [2]. In England, drug resistance to at least one of the four first line antibiotics was noted in 9.9% of culture‐positive TB cases with resistance to rifampicin in 1.9%, isoniazid in 7.3% and ethambutol in 2.0% of people with TB. MDR TB rates were 1.9% of culture confirmed cases [3]. In terms of treatment, success rates for MDR/RR‐TB remain around 60%, and hence a rapid diagnosis is important to optimise treatment and reduce the risk of developing further resistance [1].

England was the first country in the world to pioneer whole genome sequencing (WGS) on a national scale for the diagnosis of TB since 2017 [4]. Current diagnostic methods include bacterial culture followed by drug susceptibility testing (DST) either by phenotypic or genotypic methods via next‐generation sequencing (NGS) technologies. The introduction of routine WGS for all culture‐positive samples has shifted the pathway to a full genotypic strategy with phenotypic sensitivities only being performed when first line resistance is detected by WGS, or if WGS does not give complete results for first‐line drugs. Rapid polymerase chain reaction (PCR) tests may also be performed directly from clinical samples, depending on the local laboratory capacity and the clinical suspicion of infection/drug resistance.

As positive cultures are still required prior to DST (phenotypic and genotypic), the results can take days to weeks [5–7]. WGS can provide equivalent results compared to phenotypic DST with a faster turnaround time [8]. Studies have shown that WGS results can be available within 72 h of delivery of the culture sample, whilst phenotypic DST (pDST) for first‐line treatment takes an average of 28 days after culture confirmation [5]. However, in our clinical study in London, we demonstrated a real‐life and total turnaround time of 35 days from sample collection to full WGS results, when using a national centralised laboratory [7].

This highlights the need to optimise rapid diagnostic tools to firstly identify the presence of *Mycobacterium tuberculosis* (MTB) as well as test for drug susceptibilities for the initiation of appropriate treatment regimens at the earliest opportunity and to meet the WHO End TB Strategy [9]. Given the established WGS pathway in England and the time still required for the final results, alternative molecular diagnostics are being considered to further improve identification of drug‐resistant TB and the turnaround time.

The launch of Xpert MTB/XDR (Cepheid, Sunnyvale, CA, USA), which extends the range of the original GeneXpert platform (Xpert MTB/RIF, Xpert MTB/RIF Ultra), could accelerate the efforts to identify MTB and drug‐resistant TB. The GeneXpert platform can be operated with minimal technical training, and the results are available within 90 min, once the sample is placed into the machine. It can test for second‐line drug resistance, detecting mutations associated with isoniazid, fluoroquinolones, second‐line injectable drugs (amikacin, kanamycin and capreomycin) and ethionamide. Data from two multi‐centre studies from high MDR TB burden countries have shown a sensitivity of 98.9% (CI: 96.2–99.9) with a wide range in specificity of 22.5% (CI: 14.3–32.6) to 100.0% (CI: 86.3–100.0). For isoniazid resistance, the sensitivity and specificity were 94.2% (CI: 87.5–97.4) and 98.5% (CI: 92.6–99.7) against pDST. For fluoroquinolones, the sensitivity and specificity were 93.2% (88.1–96.2) and 98.0% (90.8–99.6) against pDST [10]. However, there is currently a lack of data validating the performance and clinical utility of Xpert MTB/XDR in low‐incidence countries.

Deeplex Myc‐TB (GenoScreen, Lille, France) is a targeted NGS technique that allows for deep DNA sequencing of 24‐plexed amplification of the main resistance targets, species and strain identification of MTB complex and nontuberculous mycobacteria (NTM). It can detect mutations in 18 gene regions for first‐ and second‐line drug resistance [11]. It has a reported turnaround time of around 48 h, but there are no reports of its use in routine clinical practice (outside research studies) in a low‐incidence country yet. Table 1 summarises the genotypic targets for Xpert MTB/XDR and Deeplex Myc‐TB.

**TABLE 1:** Gene targets for Xpert MTB/XDR and Deeplex Myc‐TB.

Drug	Gene target used by Xpert MTB/XDR (nucleotide interrogated)^∗^	Gene target by Deeplex Myc‐TB^∗^	Potential other gene targets not covered by both platforms
Rifampicin	N/A (separate Xpert MTB/RIF Ultra needed as initial rapid test)	rpoB	—

Isoniazid	inhA promoter (−1 to −32 intergenic)	inhA	Ndh kasA furA ^∗^10%–15% of low level INH resistance do not have mutations in the katG or inhA gene
	katG (939–957)	katG	
	fabG1 (597–630)	fabG1	
	oxyR‐ahpC intergenic region (−5 to −50 intergenic or −47–92)	aphc	

Pyrazinamide	N/A	pncA	—

Ethambutol	N/A	embB	—

Fluoroquinolones	gyrA (261–285)	gryA	—
	gyrB (1596–1632)	gryB	—

Amikacin	rrs (1396–1417)	rrs	rpsL

Kanamycin	rrs	rrs, els	

Capreomycin	rrs	rrs, tlyA	

Streptomycin	N/A	rrs, gid8, rpsL	—

Ethionamide	inhA promoter (−1 to −32 intergenic)	inhA, ethA, fabG1	—

Bedaquiline, clofazimine	N/A	rv0678	—

^∗^As per manufacturers’ datasheets.

This was a retrospective laboratory evaluation performed at University College London (UCL), UK, to analyse the diagnostic performance of the new molecular platforms Xpert MTB/XDR and the Deeplex Myc‐TB.

## 2. Methods

Twenty MTB clinical isolates (Table 2) with known drug resistance patterns confirmed by WGS and pDST at the National Mycobacterium Reference Service (NMRS) (UK Health Security Agency) were included in this study. Two control samples (including a fully susceptible MTB and *H37Rv*) and two *Mycobacterium abscessus (*subspecies *abscessus)* isolates were also used.

**TABLE 2:** List of MTB/NTM clinical isolates with resistance patterns.

Isolates	Drug susceptibility	Phenotypic resistance (associated genotypic mutations if known)
*H37Rv* ^∗^	DS WT	Fully susceptible, reference strain
2.113^#^	DS WT	Fully susceptible, used as control
2.302	DR	SM
3.039	DR	INH (inhA C ⟶ T)
2.292^∗^	DR	INH (inhA C ⟶ T)
3.303	DR	INH (inhA C ⟶ T)
4.018	DR	INH, RIF, ETH, clarithromycin (inhA C ⟶ T)
4.194	DR	INH (katG S315 T)
4.211	DR	INH (inhA C ⟶ T)
4.493	DR	INH (inhA C ⟶ T)
4.503	DR	INH (inhA C ⟶ T)
7.116^#^	DR	INH, ETH (inhA C ⟶ T)
7.118	DR	INH (katG S315 T)
5.177	DR	INH (inhA C ⟶ T)
4.011	DS	(katG R463 L, not conferring resistance)
3.313	DR	SM
333^∗^ (MDR)	MDR	INH, RIF, SM
346 (MDR)	MDR	INH, RIF, SM
401 (MDR)	MDR	RIF, INH
408^#^ (MDR)	MDR	INH, RIF, SM
421^∗^, ^#^ (XDR)	XDR	INH, RIF, SM, EMB, CAP, FQ (moxifloxacin)
433^∗^, ^#^ (XDR)	XDR	INH, RIF, SM, EMB, PZA, CAP, FQ (moxifloxacin)
T1109 (*Mycobacterium abscessus*)	DR Mab, used for coinfection experiments	AMI, ciprofloxacin, moxifloxacin
T011292 (*Mycobacterium abscessus*)	DR Mab, used for coinfection experiments	Ciprofloxacin, moxifloxacin

*Note:* XDR: extensively drug‐resistant, Mab: *Mycobacterium abscessus,* RIF: rifampicin, INH: isoniazid, PZA: pyrazinamide, EMB: ethambutol, SM: streptomycin, FQ: fluoroquinolones, KAN: kanamycin, AMI: amikacin, CAP: capreomycin, ETH: ethionamide, LIN: linezolid, BDQ: bedaquiline, and CFZ: clofazimine.

Abbreviations: DR, drug‐resistant; DS, drug susceptible; MDR, multidrug‐resistant; WT, wild type.

^∗^Samples used in the limit of detection (LOD) experiments.

^#^Samples used in the heteroresistance experiments.

Frozen clinical samples were thawed and subcultured in a liquid media Middlebrook 7H9 (Becton‐Dickinson, New Jersey, USA). These were incubated at 37°C for 7–14 days, and the turbidity of bacterial suspension was adjusted (by dilution with 7H9) to reach 0.5 McFarland (Oxoid, Hampshire, UK) to standardise the volume of starting inoculum based on previous studies, showing the conversion of McFarland to CFU [12]. This was also confirmed with an internal verification in our own laboratory by calibrating a densitometer to measure the optical density (OD) of 0.09–0.11 for an equivalent McFarland standard of 0.5 (10^7^ CFU/mL).

The Xpert MTB/XDR tests were performed as per manufacturer’s instructions in the UCL Centre for Clinical Microbiology laboratory, whilst samples for Deeplex Myc‐TB were sent directly to GenoScreen, Lille, France, following the standard GenoScreen protocol. Drug susceptibility status of all samples sent to GenoScreen was blinded for GenoScreen staff. All samples were heat killed at 95°C for 30 min prior to testing with both methods.

Four different types of experiments were designed to test the performance of the platforms:

1. Concordance of DST using Xpert MTB/XDR and Deeplex Myc‐TB in comparison to combined WGS/pDST: Each isolate was tested with both Xpert MTB/XDR and Deeplex Myc‐TB by using a fixed volume (500  μL) of the culture‐positive MTB broth after 7–14‐day incubation in 7H9.
2. Limit of detection of MTB: Five samples including isoniazid monoresistance, MDR and XDR strains (*H37Rv*, 2.292, 333, 421, 433) were used. A densitometer was used to measure the starting concentration of the original inoculum to be at 0.9 OD (equivalent to 10^7^ CFU/mL) as per previous internal verification and other authors [12]. Seven tenfold serial dilutions were made, and each dilution was tested using both platforms for each isolate.
3. Detection of coinfection using wild type (WT) MTB and 2 strains of *Mycobacterium* *abscessus* (Mab): A fully susceptible WT MTB (isolate 2.113) was mixed with two MDR Mab strains (2.113 + T1109 and 2.113 + T011292) in equal volumes (500  μL) of culture‐positive inoculum (7–14‐day incubation in 7H9) to determine the ability to detect multiple species (Deeplex Myc‐TB only) and if the coinfection with Mab altered the detection of a fully susceptible MTB strain (risk of cross‐reactivity in both platforms).
4. Detection of heteroresistance using a mixture of WT MTB and drug‐resistant MTB: A fully susceptible WT MTB (2.113) was mixed with four isolates of MDR/XDR TB (2.113 + 7.116, 2.113 + 408, 2.113 + 421, 2.113 + 433). MTB strains were initially cultured on solid 7H10 plates, and a single colony of each strain was separately subbed into liquid media to produce a turbidity of 0.9 OD. Different mixtures of 500 μL were then created by adding the WT strain to different percentages of the MDR/XDR strains. 1%, 5%, 10%, 50% and 100% of the admix MDR/XDR isolates were analysed to determine if heteroresistance was detectable using Xpert MTB/XDR and Deeplex Myc‐TB platforms.

## 3. Results

### 3.1. Concordance of DST Using Xpert MTB/XDR and Deeplex Myc‐TB in Comparison to WGS/pDST

**FIGURE 1:**
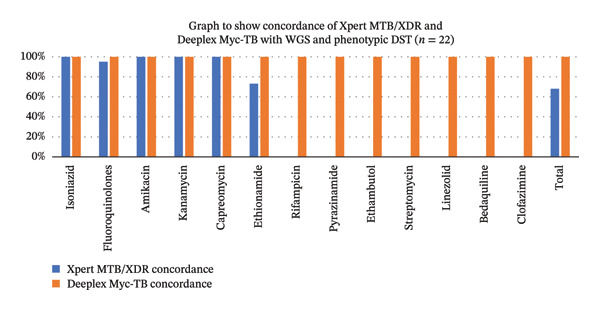
Graphical representation showing concordance of Xpert MTB/XDR (blue) and Deeplex Myc‐TB (red) when compared to WGS and pDST (routine standard) in identifying drug susceptibilities. *X* axis showing different drug susceptibilities; *y* axis showing the percentage of concordance. Xpert MTB/XDR had a total concordance of 68%, whereas Deeplex Myc‐TB was 100% concordant.

Xpert MTB/XDR had a total concordance of 68% (7/22) when compared to WGS/pDST. There was a 5% (1/22) discordance with fluoroquinolone susceptibility, with one case showing a false positive reading. Ethionamide susceptibilities had a 27% (6/22) discordance compared to WGS/pDST and were all false negative readings. All isoniazid readouts were concordant on Xpert MTB/XDR. Deeplex Myc‐TB was 100% concordant with WGS/pDST in all tests performed. This can be seen in Figure 1.

### 3.2. Limit of Detection of MTB

**FIGURE 2:**
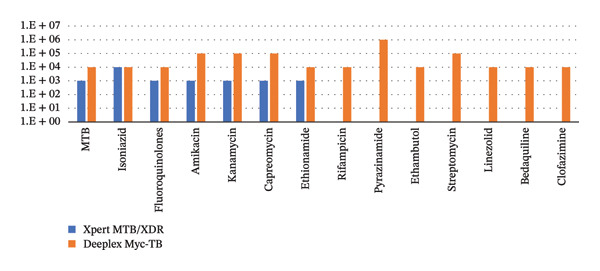
Graph showing the limit of detection of MTB and correctly identified drug susceptibilities using Xpert MTB/MDR (blue) and Deeplex Myc‐TB (red) in comparison to WGS/pDST. *X* axis showing detection of MTB and different drug susceptibilities correctly identified and *y* axis showing serial dilution in CFU/mL. Xpert MTB/XDR was able to detect all drug susceptibilities correctly at 10^4 CFU/mL, whereas for Deeplex Myc‐TB, this was at 10^6 CFU/mL.

Xpert MTB/XDR detected MTB down to 10^3 CFU/mL in all 5 isolates tested and further down to 10^1 CFU/mL in 2/5 samples (H37Rv and 2.292). Deeplex Myc‐TB detected MTB in all cases at 10^4 CFU/mL and was also able to detect MTB down to 10^1 CFU/mL in 1/5 strain (H37Rv). Xpert MTB/XDR had a limit of detection of 10^3 CFU/mL for all drug susceptibility reads to be correctly identified when compared to WGS/pDST other than for isoniazid, where 10^4 CFU/mL was required. For Deeplex Myc‐TB, a minimum of 10^4 CFU/mL was required to correctly identify drug susceptibilities for isoniazid, ethambutol, fluoroquinolones, ethionamide, rifampicin, linezolid, bedaquiline and clofazimine. For pyrazinamide readouts, there were 2 false positive readings (compared to the NMRS results) (2.292 at 10^4 CFU/mL and 421 at 10^5 CFU/mL), and hence a minimum of 10^6 CFU/mL was required for an accurate reading across all 5 isolates. For amikacin, capreomycin, kanamycin and streptomycin, there were low coverage readouts at 10^4 CFU/mL, and hence a minimum of 10^5 CFU/mL was required. The results are summarised in Figure 2.

### 3.3. Detection of Coinfection Using a Mixture of WT MTB and 2 Strains of MDR Mab

Xpert MTB/XDR results showed no cross‐reactivity with the 2 MDR Mab strains. Deeplex Myc‐TB detected WT MTB in both coinfected samples, but it was only able to detect the presence of Mab (2.113 + T011292) in one of the two samples.

### 3.4. Detection of Heteroresistance Using WT MTB and Drug‐Resistant MTB

**FIGURE 3:**
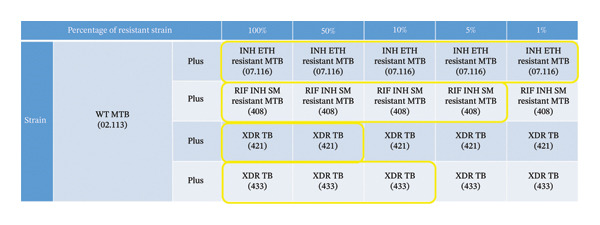
Figure showing detection of heteroresistance with Deeplex Myc‐TB using WT MTB (2.113) and 4 resistant MTB strains at different percentages. The yellow box indicates the percentage of the admix of resistant strain at which the resistant patterns were correctly identified in comparison to WGS/pDST. Deeplex Myc‐TB was able to detect down to 1% in one strain (2.113 + 07.116) but required a minimum of 50% admix for all the resistance patterns to be correctly identified in all 4 heteroresistant samples.

**FIGURE 4:**
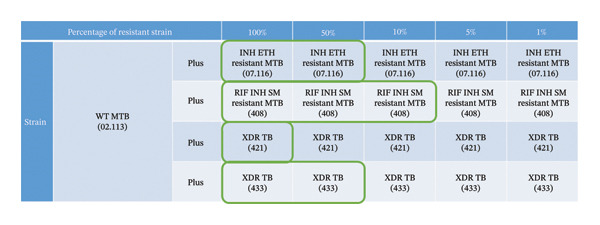
Figure showing detection of heteroresistance with Xpert MTB/XDR using WT MTB (2.113) and 4 resistant MTB strains at different percentages. The green box indicates the percentage of the admix of resistant strain at which the resistant patterns were correctly identified in comparison to WGS/pDST. Xpert MTB/XDR was able to detect down to 10% in one strain (2.113 + 408) but required 100% admix for all the resistance patterns to be correctly identified in all 4 heteroresistant samples.

Deeplex Myc‐TB required at least 50% admix of the resistant strain to accurately identify correct resistant patterns in all 4 heteroresistant samples. For the 2 non‐XDR isolates, Deeplex Myc‐TB was able to detect down to 1% (2.113 + 07.116) and 5% (2.113 + 408) (Figure 3). For Xpert MTB/XDR, 100% admix concentration was required to correctly identify all the resistant patterns in all 4 heteroresistant samples. It was able to detect down to 10% for one isolate (2.113 + 408) and 50% in the other 2 isolates including one XDR strain (2.113 + 433) (Figure 4). Of note, there was no cross‐reaction between a streptomycin‐resistant organism and other aminoglycosides.

## 4. Discussion

This study directly compared two novel diagnostic tests already CE marked and commercially available in the EU but not widely adopted into routine clinical practice yet. For the detection of MTB, Xpert MTB/XDR had a lower limit of detection at 10^3 CFU/mL compared to Deeplex Myc‐TB, where a bacteria load of 10^4 CFU/mL was needed. Both platforms are less sensitive compared to a liquid culture method (i.e., BACTEC/MGIT system by BD, USA), which can detect MTB down to 10^2 CFU/mL [13], but have a rapid turnaround time. In practice, Xpert MTB/XDR would likely be used as a reflex test, meaning the initial detection of MTB and presence of rifampicin resistance would be identified on either Xpert MTB/RIF or Xpert MTB/Ultra which has a lower limit of detection in comparison to the Xpert MTB/XDR at 10^2 CFU/mL and 156 CFU/mL, respectively [14].

With regard to DST, Deeplex Myc‐TB had a 100% concordance with WGS/pDST which is currently used for routine care in England. As an additional advantage, Deeplex Myc‐TB has a cloud‐based system where gene mutations are regularly updated by the company and aligned with the WHO Catalogue [15], plus some other relevant mutations. It can be performed directly from clinical samples (albeit not assessed in this study) or from cultured isolates, and it has an expected turnaround time of 48 h, once the sample or the culture is received in the laboratory. In contrast, Xpert MTB/XDR has preset gene targets for the detection of drug resistance, so any mutation outside these targets or certain new mutations may lead to incorrect results as previously described for the Xpert MTB/RIF [16]. Xpert MTB/XDR had some discordant results for ethionamide, but more importantly a false positive readout for fluoroquinolone due to a common mutation on *gyrA* (A90 V) not conferring resistance to the entire class [17]. Caution should be taken in the clinical context as this may inappropriately suggest modifying and unnecessarily prolonging TB treatment. Xpert MTB/XDR had a 100% concordance for isoniazid susceptibility testing, highlighting its utility as a rapid screening tool for isoniazid monoresistance (in countries with higher incidence of monoresistance).

Overall, Xpert MTB/XDR had a lower limit of detection for correctly identifying drug susceptibilities (as per WGS/pDST) in comparison to Deeplex Myc‐TB. However, Xpert MTB/XDR has a limited drug panel, now also less relevant since the WHO MDR TB treatment regimen has been updated and avoids injectables [18]. When considering current drug‐resistant regimens, at 10^4 CFU/mL, Deeplex Myc‐TB was able to identify drugs susceptibilities for fluoroquinolones, bedaquiline, linezolid and clofazimine, some of the key routinely used drugs for MDR/XDR TB treatment. However, more studies are needed to assess the correlation of the genetic mutations with clinical resistance and their comparison with pDST.

There is still limited understanding of heteroresistance and coresistance in the clinical context [19]. The accuracy of both Xpert MDT/XDR and Deeplex Myc‐TB was affected with heteroresistant populations, more so for Xpert MTB/XDR, but neither were affected by coinfections with a drug‐resistant Mab. Deeplex Myc‐TB was also able to identify one of the subpopulations of Mab. Further comparison between the two methods should also include costs, but this was not in the scope of this study and, inevitably, NGS techniques are likely to be more expensive (including capital investment and reagents) than the GeneXpert platform.

Currently, in high‐resource low‐burden settings, a combination of culture and molecular methods is often used to rapidly identify MTB and initial rifampicin resistance, which is used as a surrogate marker for MDR TB. The above two platforms have both individual advantages which could optimise early identification of drug‐resistant TB. Xpert MTB/XDR could be used to rapidly identify isoniazid monoresistance as this would allow the correct WHO drug regimen to be started immediately, which is particularly relevant in those settings with high INH‐resistance rates [3, 20, 21]. Deeplex Myc‐TB with a reported turnaround time of 48 h could offer a more comprehensive DST for MDR/XDR TB and could be used in suspected drug‐resistant cases, once identified initially using the GeneXpert platform and where the bacterial load (or CFU/mL) would allow a good performance. Many microbiology laboratories in high‐income countries have significantly increased their sequencing capacity after the COVID‐19 pandemic, by repurposing their sequencing machines originally procured for COVID‐19 sequencing and by expanding their capacity to perform WGS on bacterial isolates.

We recognise that this evaluation has several limitations. The modest number of isolates is consistent with its exploratory laboratory scope and was influenced by practical considerations, notably the high cost of the assays and the restricted funding available for this initial phase. In particular, only two fluoroquinolone‐resistant isolates were included, which limits the strength of conclusions regarding assay performance for FQ resistance and highlights the need for studies including a larger panel of FQ‐resistant and XDR strains. In addition, the study did not assess performance directly on clinical samples and did not include a formal cost analysis. In the limit of detection experiments, only five isolates were tested, and some variability was observed: this difference may reflect strain‐specific factors such as bacterial clumping, differences in DNA release during heat inactivation or minor variations in inoculum preparation and amplification efficiency.

The intention of this initial evaluation was not to provide a comprehensive assessment of the clinical utility, but rather to generate practical preliminary data on the limit of detection, coinfection and heteroresistance and to compare these findings across the two platforms. Larger prospective studies performed directly on clinical specimens will be important to further validate these results. With new molecular techniques on the horizon, there are still numerous ways to optimise TB diagnostics with faster identification of the most up to date accurate drug resistance patterns to establish correct treatment regimens to improve patient outcomes. Both Xpert MDT/XDR and Deeplex Myc‐TB represent an attractive option for the rapid detection of drug resistance whilst providing extra clinically relevant information in a variety of high‐ and low‐incidence settings.

## Funding

The authors would like to acknowledge Cepheid (Sunnyvale, California, USA) for providing all the reagents and the lease of the machine for free for the duration of the study and GenoScreen (Lille, France) for performing the tests at a discounted price and for providing initial input in the protocol design.

## Ethics Statement

No ethical approval was required for this review, but a departmental proposal was submitted and approved as per UCL policy.

## Conflicts of Interest

The authors declare no conflicts of interest.

## Data Availability

The data that support the findings of this study are available from the corresponding author upon reasonable request.
